# Antibiotic drug tigecycline inhibits melanoma progression and metastasis in a p21CIP1/Waf1-dependent manner

**DOI:** 10.18632/oncotarget.6419

**Published:** 2015-11-28

**Authors:** Huanrong Hu, Zhen Dong, Peng Tan, Yanli Zhang, Lichao Liu, Liqun Yang, Yaling Liu, Hongjuan Cui

**Affiliations:** ^1^ Department of Dermatology, The Third Hospital of Hebei Medical University, Shijiazhuang, 050000, P.R. China; ^2^ State Key Laboratory of Silkworm Genome Biology, Southwest University, Chongqing, 400715, P.R. China

**Keywords:** tigecycline, melanoma, p21CIP1/Waf1, cell growth and proliferation, cell migration and invasion

## Abstract

Antibiotics are common durgs with low toxicity but high effectiveness. They have been suggested to be drug candidates for cancer therapy in recent years. Here, we tried to investigate the antitumour effect of tigecycline on malignant melanoma. We showed that tigecycline dramatically inhibited cell proliferation and induced cell cycle arrest at G0/G1 phase. At the same time, tigecycline suppressed cell invasion and migration through preventing epithelial-mesenchymal transition (EMT) process. In addition, tigecycline also significantly blocked tumor growth *in vivo*. Expression of cell cycle-related proteins were investigated and resulted in downregulation of G1/S checkpoint proteins, such as CDK2 and Cyclin E. However, cyclin-dependent kinase inhibitor 1 (CDKN1A, p21^CIP1/Waf1^) was downregulated after tigecycline treatment, which was not conformed to its conventional function. To explain this, we overexpressed p21 in melanoma cells. We found that p21 overexpression significantly rescued tigecycline-induced cell proliferation inhibition as well as migration and invasion suppression. Taken together, our results revealed that the essential role of p21 in the inhibitory effect of tigecycline on proliferation, migration and invasion of melanoma. Tigecycline might act as a candidate therapeutic drug for treatment of patients suffering from malignant melanoma.

## INTRODUCTION

Malignant melanoma (MM) is a kind of highly aggressive dermatological malignancy with a poor prognosis. Over the past decades, the incidence of malignant melanoma is increasing [1]. According to 2013 Surveillance, Epidemiology, and End Results data, its average incidence rate rose 2.6% each year for the last decade [2]. It is considered that metastasis of melanoma has contributed to the rising morbidity and increasing mortality of skin neoplasms. And even in patients with thin small primary tumors, metastasis of melanoma occurs [3]. Until recently, despite tremendous advances have been made in multimodality therapies including surgery, radiation and chemotherapy, the prognosis for malignant metastatic melanoma remains extremely poor [4]. Therefore, it is time to investigate some novel drugs with high efficiency and minimal toxicity for metastatic melanoma. Identification of effective strategies for the treatment of metastatic melanoma remains an urgent need.

Recent reports showed that some antibiotics can repurpose for anti-cancer therapy [5], especially tetracycline and their derivatives, such as minocycline and doxycycline [6–8]. The mechanism of actions (MOAs) of these tetracyclines in tumors are independent of their antibacterial activity and showed diverse in different types of tumors. For example, glioma growth inhibition by minocycline was mediated through endoplasmic reticulum stress-induced apoptosis and autophagy [9, 10]. But in ovarian cancer, minocycline attenuated pro-oncogenic factor Hif-1α expression through modulation of p53 and AKT/mTOR/p70S6K/4E-BP1 pathway or suppresses interleukine-6, its receptor system and signaling pathways [7, 8]. Another tetracycline, doxycycline, down-regulated DNA-PK, an essential enzyme in DNA-repair, and radiosensitized tumor initiating cells [6]. In hepatocellular carcinoma, doxycycline inhibited the epithelial-to-mesenchymal transition (EMT) and vasculogenic mimicry [11]. And doxycycline combined with aspirin, lysine and mifepristone can effectively and safely prevented cancer metastasis [12]. Importantly, doxycycline induced mitochondrial membrane potential change and apoptosis of melanoma through ROS-ASK1-JNK pathway [13]. Besides, doxycycline induced apoptosis and inhibits proliferation and invasion of cervical carcinoma stem cells [14]. Above all, preclinical study showed that minocycline combinated with sabutoclax were highly cytotoxic to pancreatic cancer cells and safely efficacious *in vivo* [15]. These evidences strongly indicated that tetracyclines were effective candidates for tumor treatment.

As one of the third-generation tetracycline antibiotic, tigecycline is a derivative of minocycline with a t-butylglycylamido group instead of the hydrogen at position nine [16]. And it is approved for antibacterial treatment in clinic by FDA in 2005 [17]. It has potential activity to treat a wide variety of gram-positive and gram-negative pathogens, including multidrug-resistant strains [18, 19]. Tigecycline is a protein synthesis inhibitor by binding to the 30S bacterial ribosomal subunit. It prevents bacterial protein synthesis through inhibiting the binding of a given aminoacyl-tRNA to the A-site of the ribosome [19]. Recent reports have shown that tigecycline had antitumoral activity in acute myeloid leukemia and other 8 cancer types by inhibition of mitochondrial translation or biogenesis [5, 20]. In gastric cancer, tigecycline inhibited cell proliferation and inducing autophagy [21]. Importantly, tigecycline is non-toxic for normal cells [5]. However, the effects of tigecycline in melanoma cells are less well studied.

In this paper, we deliberated on the function of tigecycline in human melanoma progression and metastasis. Our studies first put forward that tigecycline has anti-melanoma activity through inducing proliferation inhibition, cell cycle arrest and migration/invasion suppression by downregulating p21. Tigecycline can act as a candidate agent in the treatment of metastatic melanoma.

## RESULTS

### Tigecycline inhibited cell growth and proliferation in human melanoma cells

**Figure 1:**
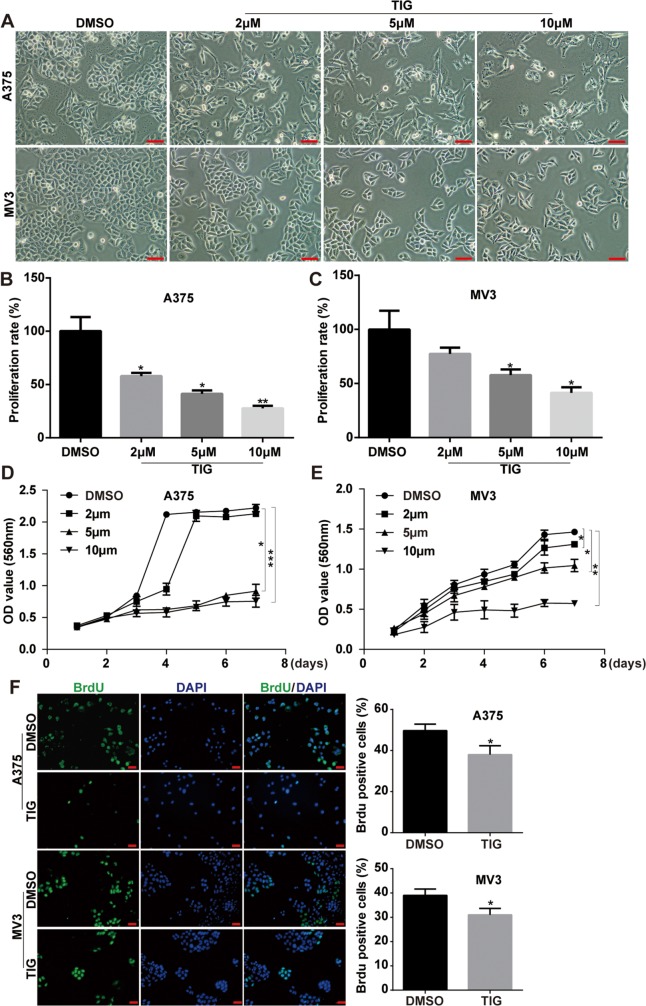
Tigcycline inhibited cell growth and proliferation in human melanoma cells **A.** Cell morphology of A375 and MV3 melanoma cells after treating with DMSO or the indicated concentration of tigecycline for 48 h, Scale bar, 100 μm. **B, C.** The effect of tigecycline on the proliferation rate of A375 and MV3 cells. **D, E.** The effect of tigecycline on the viability of A375 and MV3 cells. **F.** Image and quantification of A375 and MV3 cells positive for Brdu staining after treating with DMSO or 10 μM tigecycline for 24 h, Scale bar, 100 μm. All data are shown as the mean ± SD. Student's *t*-test was carried out. *p < 0.05, **p < 0.01, ***p < 0.001.

To assess the effect of tigecycline in proliferation inhibition, different concentration of tigecycline were treated in human melanoma A375 and MV3 cells. MTT and Brdu assay were employed. Under the microscope, cells was treated with different concentrations of tigecycline for 48 h, resulted in cell proliferation inhibition in a dose-dependent manner (Figure 1A, 1B and 1C). Then we tested the cell viability by MTT assay after 6 different dose of TIG treatment for 48 h *in vitro* and the results showed that the IC50 of tigecycline in inhibition of cell proliferation of A375 and MV3 is 7.24 uM and 10.90 uM, respectively (Supplemental Figure 1A and 1B). We futher investigated cell growth curve by MTT assay for 7 days after the addition of tigecycline (Figure 1D, 1E). The results showed tigecycline at 5 μM and 10 μM dramatically decrease cell proliferation. Brdu staining assay also showed that 10 μM tigecycline treatment for 48 h resulted in a significant decrease in the percentage of Brdu-positive cells compared to DMSO-treated cells (Figure 1F). These results demonstrated that tigecycline dramatically inhibited cell growth and proliferation in human melanoma cells.

### Tigecycline induced cell cycle arrest at G1 phase in human melanoma cells

**Figure 2:**
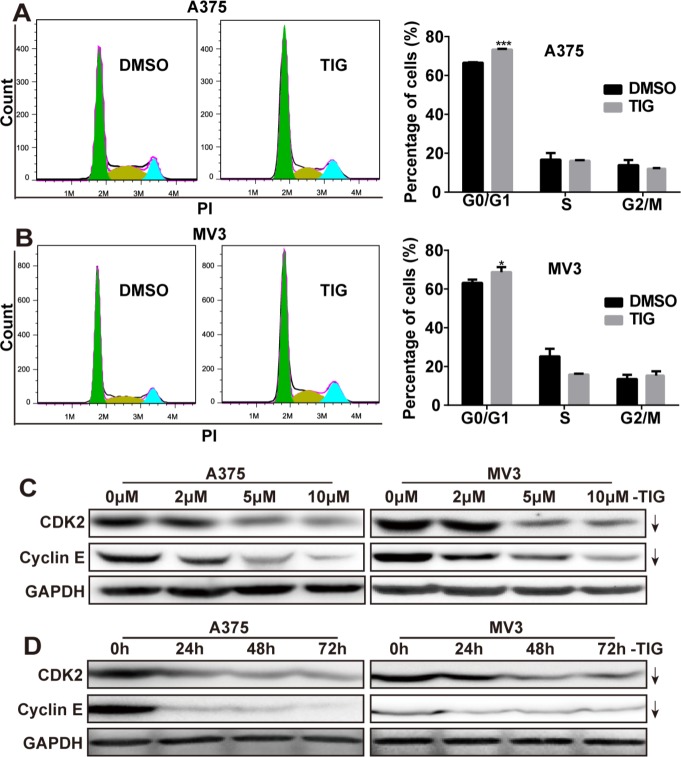
Tigecycline induced cell cycle arrest at G1 phase in human melanoma cells **A, B.** The cell cycle of A375 and MV3 cells was analyzed by flow cytometry after treating with DMSO or 10 μM tigecycline for 48 h. **C, D.** Western blot assay was performed to assess the cell cycle-related protein levels at 48 h in A375 and MV3 cells, respectively. Cells were treated with the indicated concentration or the indicated times of tigecycline; GAPDH was used as a control. All data are shown as the mean ± SD. Student's *t*-test was carried out. *p < 0.05, **p < 0.01, ***p < 0.001.

Since cell proliferation is usually regulated by the cell cycle progression, the A375 and MV3 cells were stained with propidium iodine (PI). Then the cell cycles were analyzed by flow cytometry to investigate whether tigecycline inhibited cell proliferation. Representative histograms and the results showed that tigecycline-treated cells resulted into a remarkable G1 phase arrest in A375 and MV3 cells, compared with the control cells (Figure 2A and 2B). The results demonstrated that tigecycline induced cell cycle arrest at G1 phase. To affirm the results, we measured the expression of CDK2 and Cyclin E which could promote cells to go through the G1/S checkpoint by Western blot. We found that the expression levels of cyclin E and CDK2 were decreased in tigecycline treated cells in a dose- and time-dependent manner (Figure 2C and 2D). Besides, we also checked other CDKs and cyclins and the results showed that there was no significant change of CDK4 expression, while p27, CDK6, and cyclin A and B1 were downregulated and cyclinD1 also slightly upregulated (Supplemental Figure 2A). These results suggested that tigecycline induced cell cycle arrest in human melanoma cells. All these results suggested that tigecycline-induced cell cycle arrest at G1 phase.

### Tigecycline inhibited cell migration and invasion in human melanoma cells

**Figure 3:**
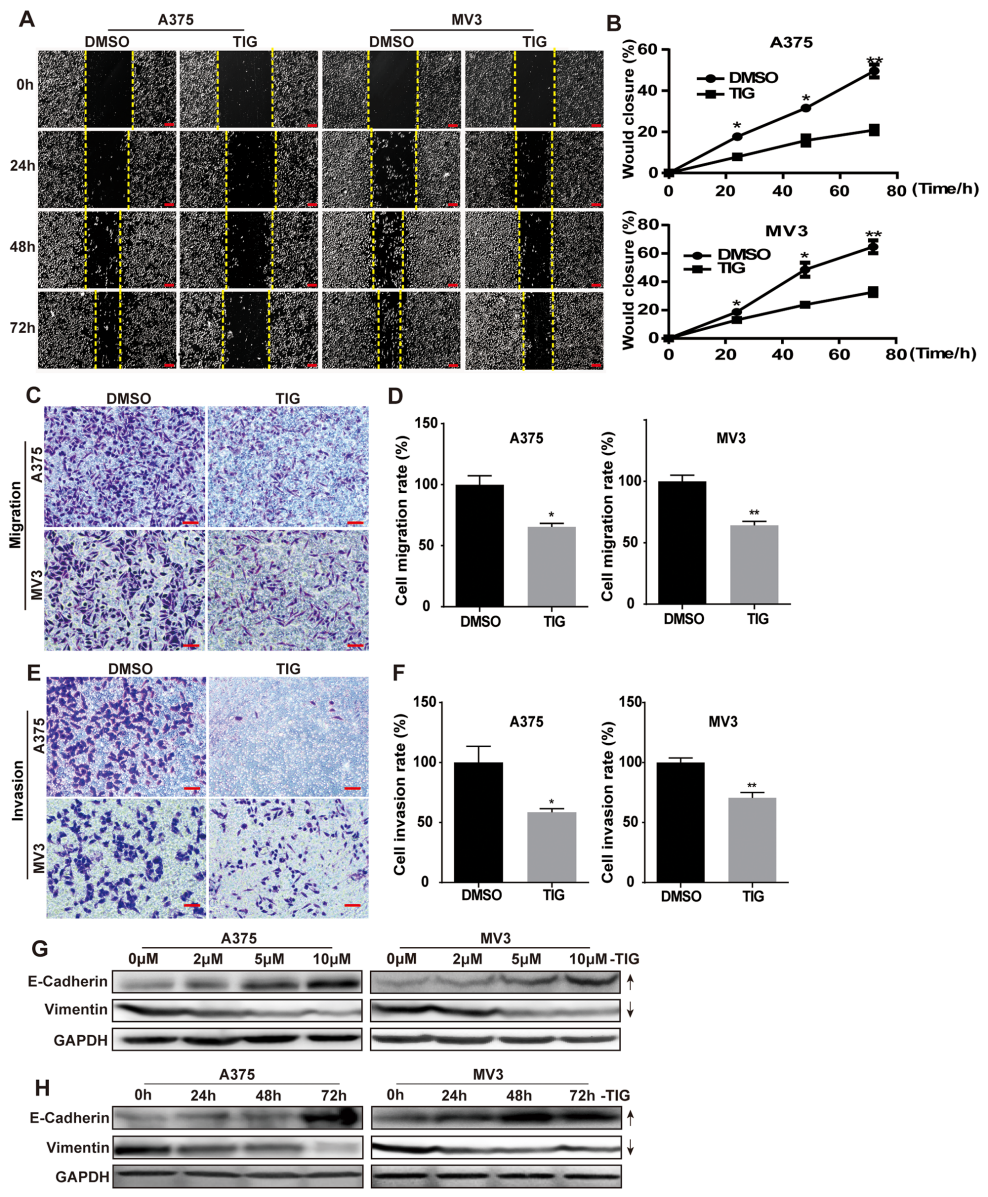
Tigecycline inhibited cell migration and invasion in human melanoma cells **A.** The migration by wound-healing assay of A375 and MV3 cells after treating with DMSO or 10 μM tigcycline for the indicated time, Scale bar, 100 μm. **B.** The effect of tigecycline on the wound closure in A375 and MV3 cells. **C.** The effect of transwell migration assays in A375 and MV3 cells after treating with DMSO or 10 μM tigcycline for 24 h, Scale bar, 100 μm. Migration rates were normalized by proliferation. **D.** The effect of transwell invasion assays in A375 and MV3 cells after treating with DMSO or 10 μM tigcycline for 72 h, Scale bar, 100 μm. Invasion rates were normalized by proliferation. **E, F.** Western blot analysis of the EMT-related protein levels at 48 h in A375 and MV3 cells respectively. Cells were treated with the indicated concentration or the indicated times of tigecycline; GAPDH was used as a control. All data are shown as the mean ± SD. Student's *t*-test was carried out. *p < 0.05, **p < 0.01, ***p < 0.001.

As metastasis is an important feature of melanoma, we next explored the function of tigecycline in migration and invasion of human melanoma cells. Cell migration and invasion abilities were evaluated by wound healing assay, transwell migration, invasion assay and Western blot. Wound healing assay revealed that cells treated with tigecycline significantly decreased the rate of lateral migration into a wound introduced in a confluent monolayer of cells compared with control groups (Figure 3A and 3B). Consistently, transwell migration assay also showed that cells after tigecycline treatment exerted seriously inbibition of the cellular transmigration ability compared with controls (Figure 3C and 3D). In transwell invasion assay, we futher verified that tigecycline treatment significantly decreased the number of cells that penetrated through the Matrigel-coated membrane (Figure 3E and 3F). Consistent with above, western blot showed that tigecycline down-regulated the expression of vimentin, a mesenchymal marker. Meanwhile tigecycline up-regulated the expression of E-cadherin, an epithelial marker, in a dose-and time-dependent manner (Figure 3E, 3F). These results indicated that tigecycline treatment reversed the epithelial-mesenchymal transition (EMT) of melanoma cells. Therefore, these results strongly demonstrated that tigecycline-induced migration and invasiveness inhibition.

### Tigecycline suppressed tumor growth in xenograft model of human melanoma cells

**Figure 4:**
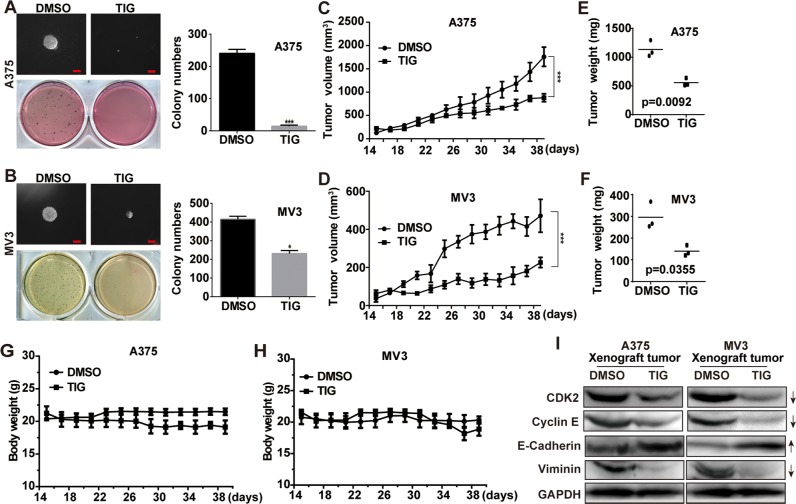
Tigecycline suppressed tumor growth in xenograft model of human melanoma cells **A, B.** The colony formation was examined by soft agar assay (1000 cells/well) in A375 or MV3 cells after treating with DMSO or 10 μM tigecycline for 14 to 21 days, Scale bar, 100 μm. **C, D.** Human melanoma cell A375 and MV3 were injected into the flank of BALA/c nude mice. When tumors were palpable, mice were treated with tigecycline (100 mg/kg) or DMSO every two days for 5 times, and tumor volume was measured. **E, F.** The weight of xenograft tumors formed by the A375 and MV3 cells which were subsquently treating with tigecycline (100 mg/kg) or DMSO. **G, H.** The weight of mice was measured after tigecycline treatment. **I.** Western blot assay was performed to assess cell cycle and EMT-related protein levels in the xenograft tumors of A375 and MV3 melanoma cells; GAPDH was used as a control. All data are shown as the mean ± SD. Student's *t*-test was carried out. *p < 0.05, **p < 0.01, ***p < 0.001.

To further assess the effects of tigecycline in colony formation, we employed soft agar assay *in vitro*. The results showed that the colonies were smaller and lesser in tigecycline-treated cells compared with control groups (Figure 4A and 4B). Melanoma cells A375 and MV3 were transplanted subcutaneously into female BALA/c nude mice. Then the mice were injected with 100 mg/kg tigecycline every two days for 5 times after the tumor plumped. The results showed that tigecycline treatment significantly blocked tumor growth (Figure 4C, 4D). The mice were sacrificed at the termination of the experiment, and the formed tumors were excised. Similar to the above, in Figure 4E and 4F, tigecycline remarkably inhibited the tumor growth in both weight and size in nude mice. Treatment with tigecycline did not affect the weight of the mice (Figure 4G and 4H) and the appearance or behavior of the mice. To test whether the tigecycline was also associated with cell cycle-related proteins *in vivo*, the expression of CDK2 and cycline E proteins was investigated in the xenograft tumor tissues of A375 and MV3 cells. In consistent with our previous results (Figure 2C and 2D), these proteins were all markedly downregulated compared with their controls (Figure 4I). Meanwhile, we detected the expression of EMT-related proteins in tumors dissected from the mice. We found that tigecycline changed relevant protein expression (Figure 4I), which agreed with our results above (Figure 3G and 3H). These results verified that tumor growth retardation was accompanied with cell cycle arrest and EMT reversal after tigecycline treatment in xenografted melanoma cells.

### Overexpression of p21 rescued tigecycline-induced cell growth and proliferation inhibition in human melanoma cells

**Figure 5:**
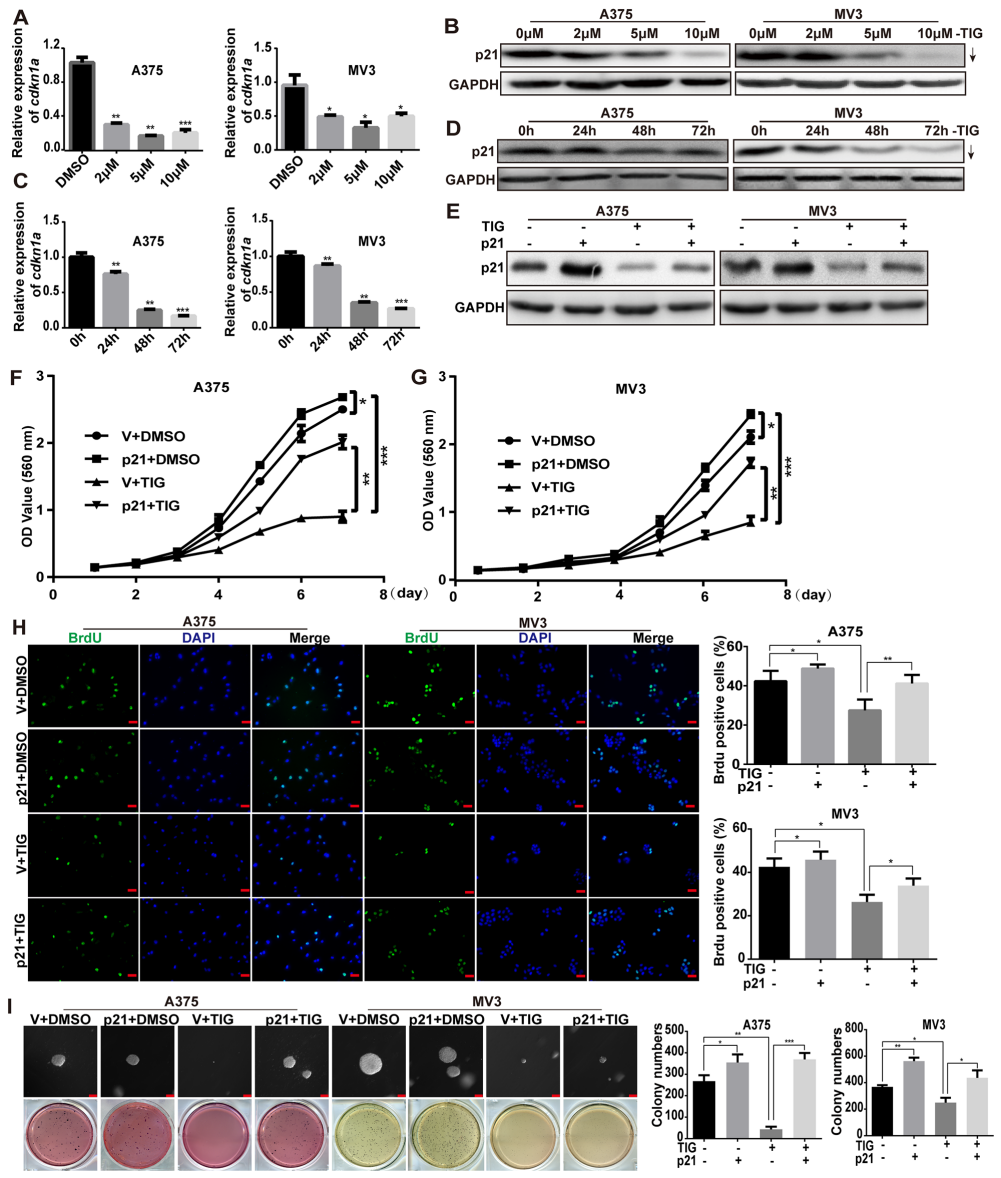
Overexpression of p21 rescued tigecycline-induced cell growth and proliferation inhibition in human melanoma cells **A, C.** RT-PCR assay was used to show the expression of *cdkn1a* (p21) mRNA after tigecycline treatment. **B, D.** Western blot assay was used to show the expression of p21 after tigecycline treatment. **E.** Western blot assay was used to show the expression of p21 in DMSO or tigecycline-treated p21/vector-overexpressed A375 and MV3 cells. **F, G.** The effect of DMSO or tigecycline on the viability of p21/vector-overexpressed A375 and MV3 cells. **H.** Image and quantification of p21-overexpressed A375 or MV3 cells as well as vector cells positive for Brdu staining after treating with DMSO or 10 μM tigcycline for 24 h, Scale bar, 100 μm. **I.** Colony formation was examined by soft agar assay (1000 cells/well) in p21-overexpressed A375 and MV3 cells as well as vector cells after treating with DMSO or 10 μM tigecycline for 14 to 21 days, Scale bar, 100 μm. Colony number was counted using counter. All data are shown as the mean ± SD. Student's *t*-test was carried out. *p < 0.05, **p < 0.01, ***p < 0.001.

During the experiments, we found that the expression of p21 was significant decreased after tigecycline treatment in a dose- and time-dependent manner, both in mRNA and protein levels (Figure 5A–5D). These results indicated that p21 may play an important role in tigecycline-induced cell growth and proliferation inhibition. A375 and MV3 cells were infected with lentiviruses encoding p21*(cdkn1a)* gene, and Western blot showed that p21 expression was upregulated after infection (Figure 5E), but the exogenous p21 expression was not decreased significantly after tigecycline treatment. These results confirmed that p21 was downregulated in mRNA level. We futher investigated cell growth curve by MTT assay for 7 days after the addition of tigecycline or DMSO in p21/vector-overexpressed A375 and MV3 cells. The results showed overexpressing p21 promoted cell proliferation, and dramatically decrease cell proliferation inhibition induced by tigecycline (Figure 5F, 5G). Brdu assay was used to examine the abilities of cell proliferation. The results revealed that the proliferation ability was rescued after p21 overexpressing in tigecycline-treated cells compared with tigecycline-treated vector cells (Figure 5H). The results were futher confirmed by soft agar assay in A375 and MV3 cells. Compared to tigecycline-treated vector groups, p21 overexpressing groups formed more colonies in soft agar in tigecycline-treated cells, while had no significance changes compared with DMSO-treated vector groups (Figure 5I). This indicated that the ability of colony formation was also reversed after p21 overexpressed. These results indicated that the tigecycline-induced proliferation inhibition was rescued by overexpression of p21 in human melanoma.

### Overexpression of p21 retrieved tigecycline-induced cell migration and invasion suppression in human melanoma cells

**Figure 6:**
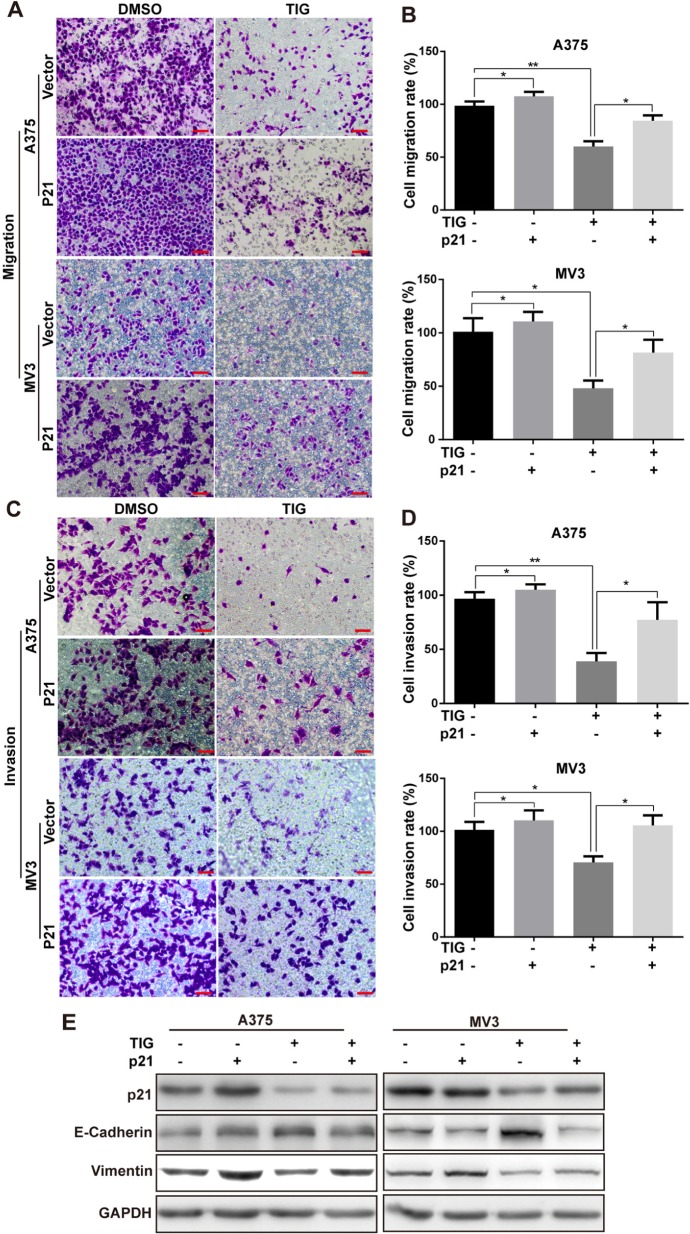
Overexpression of p21 retrieved tigecycline-induced cell migration and invasion suppression in human melanoma cells **A, B, C, D.** The effect of the transwell migration and invasion assays in p21-overexpressed A375 or MV3 cells as well as vector cells after treating with DMSO or 10 μM tigcycline for 24 h and 72 h respectively, Scale bar, 100 μm. Migration/invasion rates were normalized by proliferation. **E.** Western blot assay was used to show the expression of EMT-related proteins at 48 h in p21-overexpressed A375 and MV3 cells as well as vector cells after treated with DMSO or 10 μM tigcycline for 48 h. GAPDH was used as a control. All data are shown as the mean ± SD. Student's *t*-test was carried out. *p < 0.05, **p < 0.01, ***p < 0.001.

As p21 was reported to correlate with tumor metastasis [22], we further investigated the effects of migration and invasion in A375 and MV3 cells by overexpressing p21. Consistent to previous report [23], we found that the migration ability was partly rescued after p21 overexpressing in tigecycline-treated cells (Figure 6A and 6B), meanwhile retrieved the effects of tigecycline-induced invasion inhibition compared with tigecycline-treated vector cells (Figure 6C and 6D).

At the same time, we measured the EMT-related proteins which promoted metastatic procession. Overexpression of p21 significantly reversed the tigecycline-induced upregulation of E-cadherin and downregulation of vimentin (Figure 6E), indicating the critical role of p21 in tigecycline-induced EMT inhibition. Taken together, all the results demonstrated that tigecycline-induced metastasis inhibition in melanoma cells was retrieved after overexpressing of p21.

### Overexpression of p21 recovered tigecycline induced cell cycle arrest in human melanoma cells

**Figure 7:**
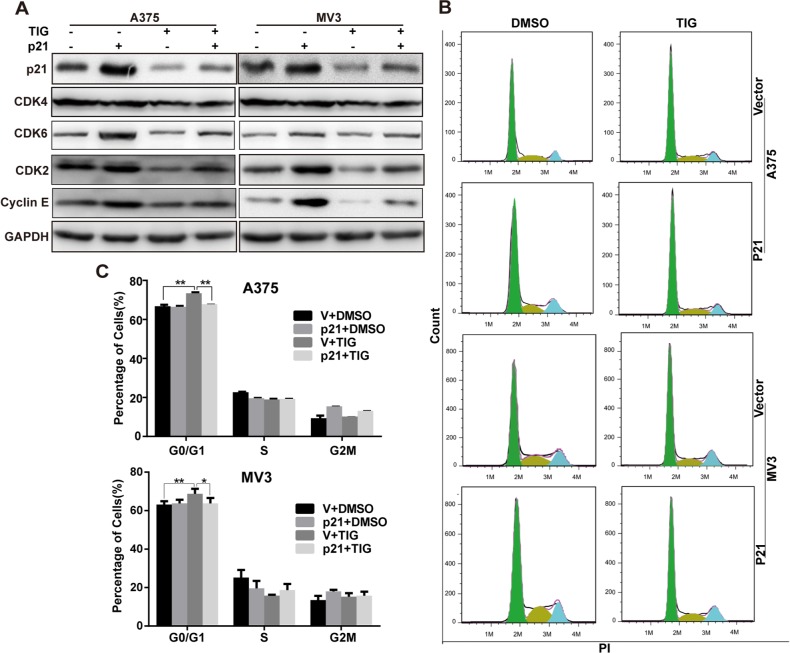
Overexpression of p21 recovered tigecycline induced cell cycle arrest in human melanoma cells **A.** Western blot analysis of cell cycle-related proteins in p21-overexpressed A375 and MV3 cells as well as vector cells after treated with DMSO or 10 μM tigcycline for 48 h. **B, C.** The cell cycle of A375 and MV3 cells was analyzed by flow cytometry in p21-overexpressed A375 and MV3 cells as well as vector cells after treated with DMSO or 10 μM tigcycline for 48 h. All data are shown as the mean ± SD. Student's *t*-test was carried out. *p < 0.05, **p < 0.01, ***p < 0.001.

Our previous results showed that p21 was decreased after tigecycline treatment (Figure 5A–5D). p21 overexpression upregulated CDK2 and cyclin E, and CDK2 and cyclin E downregulation were rescued by p21 overexpressing in tigecycline-treated cells (Figure 7A). Consistent with this, G1 arrest was also recovered by p21 overexpressing in tigecycline-treated cells (Figure 7B and 7C). That were contrary to the traditional viewpoint that p21 was a negative cell cycle controller [24, 25]. We supposed that in melanoma, p21 might be an unsual regulator, and several studies also showed p21 was a tumor activator [26–28]. One explanation is that p21 promotes CDK4/6, thus activate CDK2 [29, 30]. Our data showed that after tigecycline treatment, CDK6 expression was sharply downregulated (Supplementary Figure 2A, Figure 7A). And after overexpressing p21, the expression of CDK6 was rescued (Figure 7A). These results indicated that p21 promoted cell cycle progression in melanoma cells and overexpression of p21 recovered tigecycline induced cell cycle arrest in human melanoma cells.

## DISCUSSION

Melanoma is from the malignant proliferation of melanocytes and is the most dangerous type of skin cancer. More than 95% melanoma patients with three or more metastatic sites die within 1 year [31]. During past several decades, new targeted and immunomodulatory therapies such as vemurafenib [32] and ipilimumab [33]. They have offered some improvements in treatment of melanoma patients. Unfortunately, limited patients benefit from these therapies. Most still suffer from metastatic or relapsed disease. Tigecycline, the only FDA approved glycylcycline [17], is a third-generation tetracycline antibiotic. It is an effective chemotherapeutic agent for the treatment of patients with bacterial infection. Recent study showed that tigecycline had anti-cancer effect in multiple tumor types, including leukemia [20], gastric cancer [21] and other several tumor types [5]. However, to our knowledge, the effect of tigecycline in melanoma remains unclear.

In this study, we evaluated that the antimicrobial agent tigecycline had anti-melanoma activity. We first demonstrated that tigecycline significantly inhibited tumour growth from *in vivo* and *in vitro* experiments. MTT assays showed that tigecycline induced a dramatically decrease in cell proliferation by a dose- and time-dependent manner. Brdu assays showed a significant reduced BrdU-positive cells. Soft agar assay showed formed colonies were smaller and lesser after tigecycline treatment. Xenograft experiment showed that after tigecycline treatment, more slower and smaller tumors formed in the nude mice. The tumor weight and size is also significant reduced. All this findings together indicated that tigecycline could inhibit the growth of melanoma cells both *in vitro* and *in vivo*.

Secondarily, we found that tigecycline could strikingly inhibit melanoma cells migration or invasion by wound healing and transwell assays. Since EMT has been associated with the acquisition of migration and invasion of cancer cells [34], we detected the expression of EMT marker, E-cadherin and vimentin after tigecycline treatment. Results showed increased expression of E-cadherin, and decreased expression of vimentin. These found indicated that tigecycline inhibited EMT of melanoma cells. All evidence above showed that tigecycline inhibited migration and invision through reversing EMT progression of melanoma cells.

p21^CIP1/Waf1^, encoded by gene *cdkn1a*, was the first identified member of the cyclin-dependent kinases inhibitors (CKIs). p21 is a multifunctional protein and a key player in regulating different cellular processes, including cell proliferation, differentiation, migration, senescence, and apoptosis [35]. Conventionally, p21 has been shown to be a tumor suppressor by inhibiting cell cyle at G1 phase with suppression of cyclin E-CDK2 activity [36, 37]. However, it has been complicated by finding that p21 can exhibit oncogenic activities and p21 can exert CDK2 [23, 38, 39]. In the nucleus, p21 serves as a cell cycle inhibitor and tumor suppressor. While in the cytoplasm, it acts as an oncogene by regulating migration, apoptosis, and proliferation [40, 41]. Contrary to its conventional role in some tumors, p21 are correlated with poor prognosis, increased metastasis and high tumor grade in subsets of tumors, such as breast cancer, prostate cancer, cervical carcinomas, lymphomas as well as head and neck cancer [42, 43]. In oral melanomas, p21 expression was higher than that in intramucosal nevi [28]. Increased p21 expression levels were also found in a subset of melanomas [27]. It also has been shown that high levels of p21 in melanomas may be associated with their characteristic resistance to conventional therapies [44]. Importantly, p21 was demonstrated to protect against p53-mediated apoptosis in human melanoma cells [26]. These evidence showed that p21 may play a different role in melanoma. However, the exact mechanism has not been fully explored.

In this study, overexpression of p21 significantly rescued the tigecycline-induced proliferation inhibition, colony formation retardation, cell cycle arrest as well as migration and invasion suppression. Importantly, the expression of cell cycle and EMT-related proteins interfered by tigecycline were reversed by overexpressing p21, to some extent, at least. p21 exert CDK2/cyclin E, and promote cell cycle progression. As CDK2 and cyclin E are essential during DNA synthesis in S phase [45], BrdU incorporation reduction was also rescued after p21 overexpression. It was reported that p21 promoted CDK4/6, thus activated CDK2 [29]. Our Supplementary Data Figure 2A and Figure 7A also showed that after tigecycline treatment, CDK6 expression was sharply downregulated. And after overexpressing p21, the expression of CDK6 was rescued. Another study [27] gave a possible explanation of the tolerance of increased p21 levels found in some melanomas. They demonstrated that p21 regulated the promoter of microphthalmia-associated transcription factor (MITF), a transcription factor which plays a central role in the expression of melanocyte-specific genes, lineage determination, and survival of melanoma cells. Besides, cytoplasmic p21 could enhanced migration is associated with metastasis via the inhibition of the RhoA-pathway and a changed phosphorylation level of cofilin [46]. But how p21 overexpression exerted tumor cell aggressiveness and EMT remains a mystery. These found indicated that tigecycline inhibited proliferation, cell cycle and migration/invasion of human melanoma cell lines were p21-dependent. Clearly, much more work on tigecycline is required before we make sure the specific mechanism of p21 underlyling in melanoma and the molecular mechanism of actions of tigecycline induced p21 downregulation.

In summary, we firstly demonstrated that tigecycline induced melanoma progression and metastasis inhibition through downregulating p21, which is contrary to its conventional role. Our studies originally indicated that tigecycline may be an effective and promising therapeutic agent for melanoma treatment.

## MATERIALS AND METHODS

### Cell culture

Human melanoma cell lines (A375 and MV3) were obtained from American Type Culture Collection (ATCC, Rockville, MD, USA). Briefly, cell lines A375 was cultured in Dulbecco's modified Eagle's medium (DMEM, Life Technologies, Grand Island, NY, USA). MV3 was maintained in Roswell Park Memorial Institute-1640 (RPMI-1640; Gibco). They were supplemented with 10% fetal bovine serum (FBS; Gibco) and 1% penicillin-streptomycin (P/S). Cells were cultured at 37°C in humidified incubator with 5% CO_2_.

### Drug treatment

Tigecycline (TIG, Wyeth, Canada) was dissolved in Dimethyl Sulfoxide (DMSO) as 100 mM stock solutions. Human melanoma cell lines A375 and MV3 were treated with tigecycline at indicated concentrations or times. The cell morphology were taken by the Olympus microscopy (Olympus, Japan). Cell vitality was analyzed by Trypan blue exclusion assay.

### Cell proliferation and viability assays

Cell proliferation was determined by 3-(4,5-dimethylthiazol-2-yl)-2,5-diphenyl tetrazolium bromide (MTT) assay. Briefly, 2000 cells/well were plated onto 96-well plates then the MTT assay was carried out according to the manufacturer's protocol. After incubated with tigecycline or DMSO for the indicated time, 20 μl MTT (5 μg/ml MTT in PBS; Sigma) was added to each well, then incubated at 37°C for 2 h and removed the formazan complex. The absorbance was measured with a wavelength of 560 nm after shaking for 10 min. The experiments were carried out independently in triplicate.

### Brdu staining

Cells were cultured in 24-well plate and treated with either DMSO or tigecycilne for 24 h, incubated with 10 μg/ml thymidine analog 5-bromo-2-deoxyuridine (Brdu; Sigma) for 30 min, then washed twice with PBS, fixed in 4% paraformaldehyde (PFA) for 15 min. Afterwards, cells were permeabilized with 0.3% Triton X-100 for 5 min, pre-treated with 1 mol/L HCl for 10 min, blocked with 10% goat serum for 1 h, then a monoclonal rat primary antibody against BrdU (1:300, ab6326, Abcam, Cambridge, MA, USA) was incubated for 1 h, followed by Alexa FluorR® 594 goat anti-rat IgG secondary antibody, (H+L; Invitrogen). DAPI (300 nM) was used for nucleus staining, then the percentage of BrdU was calculated. In the end, More than 8 microscopic fields were taken (Nikon 80i, Nikon Corporation, Tokyo, Japan).

### Cell cycle assay

Cells were cultured in 6-cm dishes (3 × 10^5^ per dish) for 24 h and then treated with either 10 μM tigecycline or DMSO. After 48 h theatment, cells were washed with cold PBS, and then fixed in 70% ethanol overnight at 4°C. Subsquently, the cells were mixed with RNaseA and stained with propidium iodide (PI) (BD Biosciences, San Jose, CA, USA) at room temperature for 30 min in the dark after washing twice with PBS, followed by using a FACS C6 (BD Biosciences, San Jose, CA, USA) with CellQuest software to analysis. The data were analyzed with FlowJo7.6 software. The experiments were repeated more than three times.

### Wound-healing assays

Cells were cultured in 24-well plate and reached full of confluence, then the monolayer of the cells were scratched using a yellow pipette tip. Subsequently, PBS was used to wash and remove floating and damaged cells, and serum-free medium with 10 μM tigecycline or DMSO were added to cells for culture. Cells migrated over the denuded area were observed and pictures were taken at indicated times. The corporation of would closure was measured at the indicated time points.

### Cell migration and invasion

The migration assay was conducted in a 24-well transwell cell culture apparatus fitted with multiporous polycarbonate membrane insert (8-μm pore size) (Corning). Briefly, 2 × 10^5^ cells were seeded into the upper well of the insert with 200 μl media of 1% serum, 500 μl media contained 10% FBS were added as a chemoattractant. All media contains 10 μM tigecycline or DMSO. After incubation at 37°C in 5% CO_2_ incubator for 24 h, the chamber were rinsed three times with PBS, nonmigrating cells from the top wells were removed by cotton swabs. Cells on the lower membrane surface were fixed with 4% paraformaldehyde and stained with 0.5% crystal violet. Migrating cells was observered by 560 nm absorbance after blenched with 33% acetic acid. The transwell invasion assays were done under the same conditions as the transwell migration assays, but the top chamber of transwell were coated by 50 ul Matrigel (10 mg/mL, Corning) with 2 × 10^4^ cells/ml and the lower chamber with 20% FBS, then incubated for 72 h. Each experiment was performed in triplicate and migration/invasion rate was normalized by proliferation rate.

### Western blot assay

A375 and MV3 melanoma cell lines were harvested, then suspended in RIPA Lysis Buffer. Protein concentrations were detected with BCA protein assay kit (Beyotime Biotech, China). The lysates from cells as well as the fresh tissues were separated by SDS-PAGE, followed by transferring onto PVDF membranes (Millipore, USA). Followed by blocking with 5% BSA, the PVDF membranes were incubated gently with a primary antibody against human GAPDH (1:1000, Beyotime), p21 (1:1000, Cell Signaling), CDK2 (1:1000, Cell Signaling), Cyclin E (1:1000, Cell Signaling), E-cadherin (1:1000, Cell Signaling) and vimentin (1:1000, Cell Signaling) at 4°C overnight, followed by appropriate (horseradish peroxidase-conjugated secondary antibody) HRP-conjugated secondary antibodies. HRP-labeled goat anti-mouse IgG (H + L) (A0216, 1:2000) and goat anti-rabbit IgG (H + L) (A0208 1:2000) were used as secondary antibodies which purchased from Beyotime. Proteins were visualized by ECL Western blot analysis system. The signal was captured by the ECL reagent (Beyotime) and visualized by Western blotting detection instruments (Clinx Science).

### Quantitative real-time PCR (qRT-PCR)

Total RNA was extracted using Trizol (Takara Bio, Inc., Shiga, Japan) according to the manufacturer's protocol and mRNA was reverse transcribed into cDNA using M-MLV reverse transcriptase (Promega Corporation, Madison, WI, USA). The *cdkn1a* and *cdkn1b* mRNA transcripts were determined using the SYBER Green PCR Master mix (Takara) by quantitative RT-PCR. RT-qPCR reactions in triplicate were conducted using the LightCycler96 real-time PCR system (Roche). The individual values were normalized to that of the GAPDH control. *Cdkn1a* forward primer: AGTCAGTTCCTTGTGGAGCC; reverse primer: CCGCAGAAACACCTGTGAAC.

### Soft agar colony formation assay

Colony formation ability was determined by soft agar assay on A375 and MV3 cells. Briefly, 1.5 ml DMEM or RPMI 1640 medium containing 0.6% agarose were added to each well of a six-well culture plates and allowed to solidify (base agar). 1 × 10^3^ of A375 and MV3 cells were then mixed with 1ml DMEM or RPMI 1640 medium containing 0.3% agarose and added to the top of base agar (top agar). The cells were cultured for 14 to 21 days at 37°C under 5% carbon dioxide. At the end, cells were stained with MTT, then imaged using a digital camera. Colonies with more than 50 cells were counted using the inverted microscope.

### Tumor xenografts

4 weeks old female nude mice (BALA/c), (Beijing laboratory animal research center, China) were purchased and housed in the SPF room to acclimate for a week. Melanoma cells A375 and MV3 cells (1 × 10^6^) in 100 ul DMEM or 1640 were subcutaneously injected into both flanks of each mouse respectively. After 14 days tumor growth, the mice were divided into two groups randomly. One group was injected intraperitoneally with tigecycline at 100 mg/kg (mice body weight), meanwhile, the other group was injected with DMSO as a control every two days for 5 times. Tumor growth was measured by caliper measurement daily, and tumor volume was then calculated with the formula (volume = tumor length × width^2^ × 0.5236) after tumor plumped. At the termination of the experiment, tumors were removed, weighed. All animal experiments were pre-approved by the Institutional Animal Care and Use Committees of the Southwest University.

### Vector construction and infection

Human full length p21 cDNA was obtain from NCBI (NM_000389.4), the DNA fragment was then cloned into PCDH-CMV-MCS-EF1-puro vector to generate the recombinant plasmid. The p21 overexpression vectors were transfected into 293FT cells by using the Lipofectamine 2000 reagent (Invitrogen, Carlsbad, CA, USA), subsequently, the Lentivirus were infected into Human melanoma cells according to the manufacturer's protocol. The transfected cells were selected with puromysin (4 μg/ml) for 3 days, eventually, the drug-resistant cells were gathered, expanded and identified.

### Ethics statement

This study was performed in accordance with the approved guidelines. The protocol was pre-approved by the Institutional Animal Care and Use Committees at Southwest University. All works were made to minimize the suffering of the animals.

## SUPPLEMENTARY FIGURES AND TABLES


