# Supplementary material for: Antibiotic drug tigecycline inhibits melanoma progression and metastasis in a p21CIP1/Waf1-dependent manner
Source: Oncotarget. 2015 Nov 28;7(3):3171–85. doi: 10.18632/oncotarget.6419 (PMC4823098; doi:10.18632/oncotarget.6419)
Supplement: Supplementary file 1 [file oncotarget-07-3171-s001.pdf]

SUPPLEMENTARY FIGURES

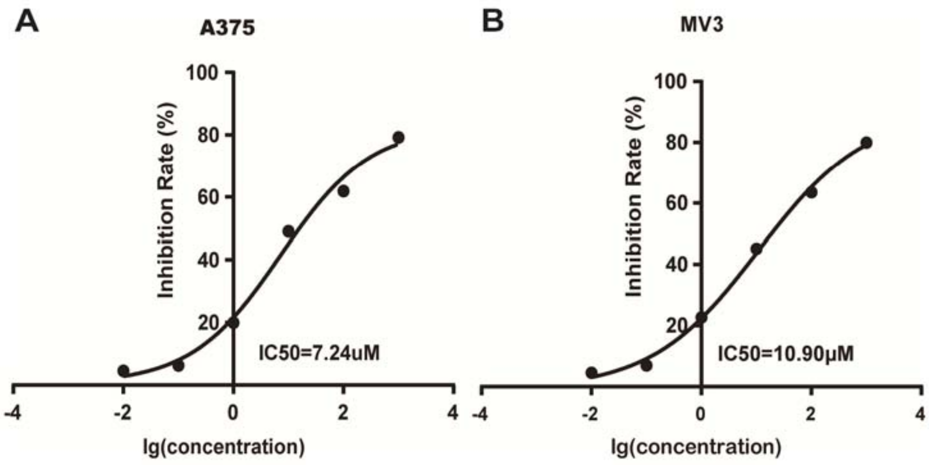

Supplementary Figure S1: A, B. IC50 of tigecycline-induced proliferation inhibition in A375 and MV3 cells. Cells were treated with 0.01, 0.1, 1, 10, 100, 1000 µM tigecycline for 48 h, and inhibition rate was analyzed by MTT assay. IC50 was calculated by GraphPad Prism 6.

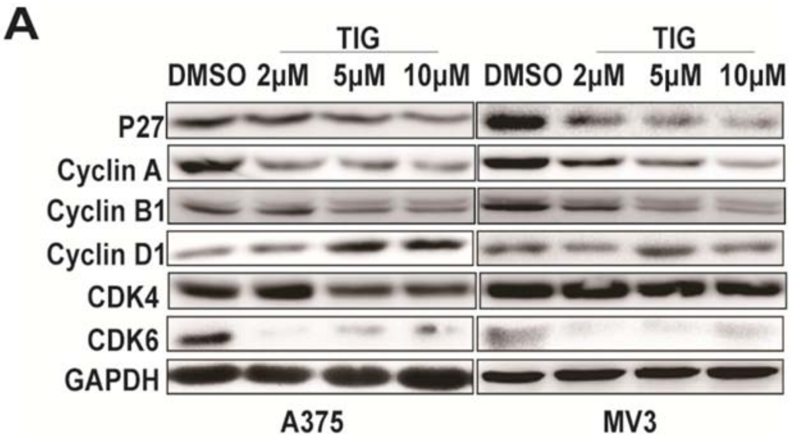

Supplementary Figure S2: A. Western blot assay was performed to assess the cell cycle-related protein levels at 48 h in A375 and MV3 cells, respectively. Cells were treated with the indicated concentration of tigecycline for 48 h; GAPDH was used as a control.
